# Red light improves spermatozoa motility and does not induce oxidative DNA damage

**DOI:** 10.1038/srep46480

**Published:** 2017-04-20

**Authors:** Daryl Preece, Kay W. Chow, Veronica Gomez-Godinez, Kyle Gustafson, Selin Esener, Nicole Ravida, Barbara Durrant, Michael W. Berns

**Affiliations:** 1 Department of NanoEngineering, University of California, San Diego, La Jolla, CA 92093, USA; 2 Department of Bioengineering, University of California, San Diego, La Jolla, CA 92093, USA; 3 Center for the Reproduction of Endangered Species, Zoological Society of San Diego, San Diego, CA 92112, USA; 4 Department of Biomedical Engineering, University of California, Irvine, Irvine, CA 92697, USA; 5 Beckman Laser Institute, University of California, Irvine, Irvine, CA 92697, USA

## Abstract

The ability to successfully fertilize ova relies upon the swimming ability of spermatozoa. Both in humans and in animals, sperm motility has been used as a metric for the viability of semen samples. Recently, several studies have examined the efficacy of low dosage red light exposure for cellular repair and increasing sperm motility. Of prime importance to the practical application of this technique is the absence of DNA damage caused by radiation exposure. In this study, we examine the effect of 633 nm coherent, red laser light on sperm motility using a novel wavelet-based algorithm that allows for direct measurement of curvilinear velocity under red light illumination. This new algorithm gives results comparable to the standard computer-assisted sperm analysis (CASA) system. We then assess the safety of red light treatment of sperm by analyzing, (1) the levels of double-strand breaks in the DNA, and (2) oxidative damage in the sperm DNA. The results demonstrate that for the parameters used there are insignificant differences in oxidative DNA damage as a result of irradiation.

Current methods of assisted reproduction in humans and animals rely on the use of drugs to increase fertility or on the direct injection of sperm into the oocyte. These methods are not always successful and could benefit from additional means of improving fertilization. Several studies have indicated that low doses of red light cause an increase in sperm motility and overall fertilizing potential in several species^1^,^2^,^3^,^4^. By increasing sperm swimming speed *in vitro*, the likelihood of contact with the oocyte is increased. Therefore, low-level light exposure has the potential to improve fertility, particularly with respect to *in-vitro* fertilization (IVF).

There have been studies characterizing the benefits of red light irradiation of sperm as well as a resulting discussion of the potential harm to sperm DNA. A recent study has shown that red light from LEDs can increase sperm quality by increasing swimming speed and mitochondrial membrane potential^3^. However, DNA damage was not tested in this the study. Several studies^2^,^3^,^5^ have also used the well-known computer-assisted sperm analysis (CASA) system for measurement of sperm motility by measuring swimming speed directly after light irradiation. Such studies assume that optically induced effects last several minutes following irradiation. Additionally, CASA uses a high-powered flash lamp to record images^6^, which may bias study results. Other studies have used light scattering to measure sperm movement^7^. However, this method samples only sperm that swim through the measurement beam.

Most computer-assisted video micrography tracking methods track only the head of the sperm and look for a bright patch of pixels which is defined as the center of the sperm cell. Under coherent light, sperm appear dark in a light background, which would affect the method of identifying sperm heads based on intensity. This method may also be problematic in the case of varying sperm morphologies such as those found in numerous animal species. Sperm also have long, flexible flagella, thus shape is not constant. Excluding the measurement of these flagella results in the loss of information about sperm movement. Also, it is pertinent that sperm illuminated by coherent radiation also generate “diffraction rings” due to the Mie scattering pattern of each cell. In addition, laser speckle may further complicate pattern recognition. While sperm are confined close to the focal region of the microscope, a full integral transform of the image is unnecessary. Our approach is to use a novel wavelet tracking algorithm which can measure sperm motility both under coherent and incoherent illumination, thus providing the ability to directly compare samples irradiated with and without the red light.

Additionally, it has been shown that 630 nm laser light stimulation can lead to the production of reactive oxygen species (ROS), such as hydrogen peroxide (H_2_O_2_) in the mitochondria of mouse sperm^4^. Low levels of ROS act as an important messenger in fertilization, allowing the sperm acrosome to react with the oocyte^8^. However, high levels of ROS can be damaging to cell DNA. Therefore, it is possible that light stimulation can cause oxidative DNA damage that may be more visible in nuclear DNA found near the sperm midpiece where the mass of cellular mitochondria are concentrated. Previous studies have shown that DNA damage can lead to infertility in men and that oxidative stress from ROS is the main cause of DNA fragmentation^9^,^10^. Therefore, careful assessment of DNA damage is important.

Oxidative DNA damage can lead to multiple forms of mutations as a result of structural alterations of bases as well as helix distortions and the production of single and double-strand DNA breaks. The most common product of oxidative DNA damage is oxidation of guanine to form 8-hydroxyguanine (8-oxo-G). If left unrepaired this base will pair with adenine instead of cytosine^11^. A previous study showed that sperm are capable of phosphorylating histone H2AX on Serine 139 in the presence of H_2_O_2_^12^. This protein modification is termed γH2AX and is used by the cell to mark double-strand breaks up to 1–2 megabases beyond the damage site. Therefore it is deemed to be a highly sensitive marker for DNA double-strand breaks produced by ROS^13^,^14^. Firestone *et al*. found that 30 seconds of irradiation with 905 nm light did not result in significant levels of DNA fragmentation as measured using acridine orange^2^. This method distinguishes single-stranded from double-stranded DNA based on fluorescence emission of sperm that have been acid treated. The presence of single-stranded DNA shifts the emission from green to red. This method has been widely used to assess DNA integrity of spermatozoa since its first description in 1980^15^. However, this method does not quantitate the level of oxidative DNA damage after laser stimulation or of the level of double-strand breaks in intact spermatozoa DNA.

Recent publications do not adequately assess the safety of 633 nm irradiation with respect to DNA damage. The proposed method of irradiation is intended for eventual use for fertilization and live birth. Therefore, we felt it was critical to test the effects of red light stimulation on sperm DNA. The visualization and localization of γH2AX would allow us to determine whether the laser or mitochondrial stimulation are causing any detectable double-strand breaks. Additionally, we tested for the presence of 8-oxo-G in control and irradiated sperm via a quantitative enzyme linked immunosorbent assay (ELISA). This sensitive assay would allow for the determination of whether there was significant oxidative DNA damage following irradiation with 633 nm light.

## Results

### Comparison of CASA speed measurements with wavelet-based algorithm measurements

**Figure 1:**
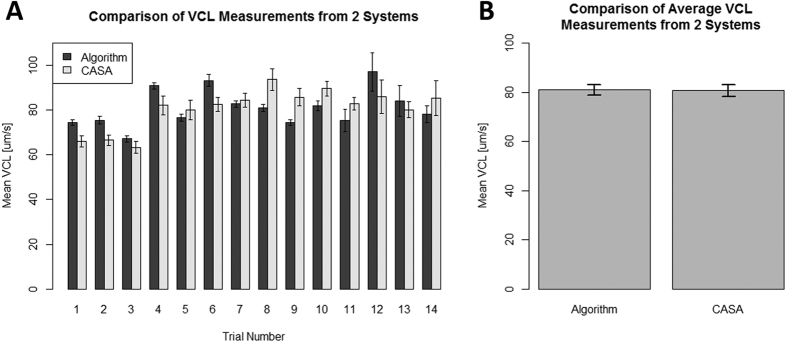
Comparison of VCL Measurements from CASA and Wavelet-Based Algorithm. Error bars in both graphs indicate standard error. (**A**) Mean VCL Measurements from 14 Trials. VCL measurements from the algorithm are multiplied by the scaling factor (1.59). (**B**) VCL Measurements Averaged Across 14 Trials. A t-test determined that there was no statistically significant difference between the two test groups (p = 0.464).

It was determined that the IVOS CASA system values for average curvilinear velocity are 1.59 ± 0.17 times the respective values of our algorithm. VCL measurements were taken using the CASA system and our algorithm. The measurements taken with our algorithm were multiplied by 1.59 to show the consistency of our measurements compared with the standard measurement (Fig. 1).

### Swim velocity following red light stimulation

**Figure 2:**
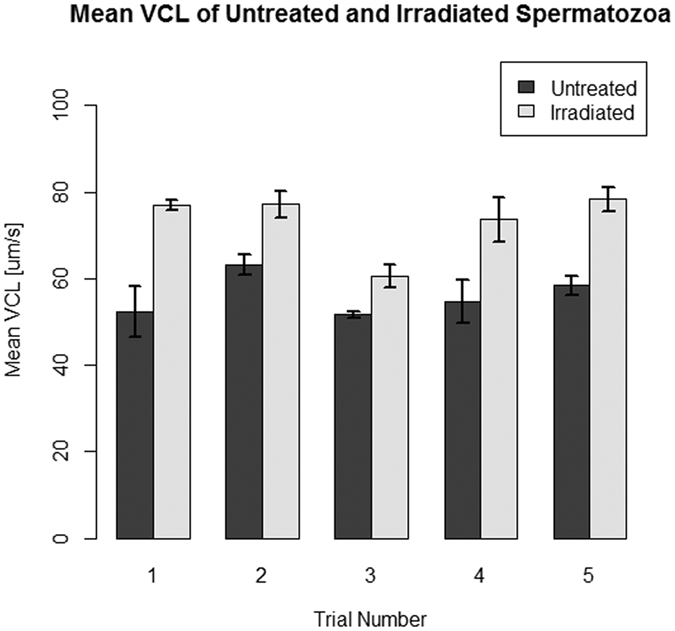
Comparison of irradiated and control sperm speeds. The average VCL was measured for sperm exposed to 633 nm light for 35 minutes and for the control group. Error bars indicate standard error. The VCLs for exposed sperm were considerably higher than those of their control counterparts. Each trial represents four measurements taken at specific time points after thawing from a sample, wherein aprox.1000 individual sperm traces were made per measurement. A t-test was performed for each trial to determine whether laser irradiation produced a statistically significant increase in swimming speed compared with the control. The p-values (denoted p_*i*_ where *i* = trial number) generated from those t-tests are p_1_ = 0.0235, p_2_ = 0.00667, p_3_ = 0.0181, p_4_ = 0.0139, and p_5_ = 0.000161.

All samples exposed to the laser exhibited higher average curvilinear velocity (VCL) measurements than their control counterparts by 17–47% (Fig. 2). Increased speed persisted throughout irradiation time. Increases in swimming speed were observed within 35 minutes of irradiation.

### Assessment of DNA damage following red light stimulation

**Figure 3:**
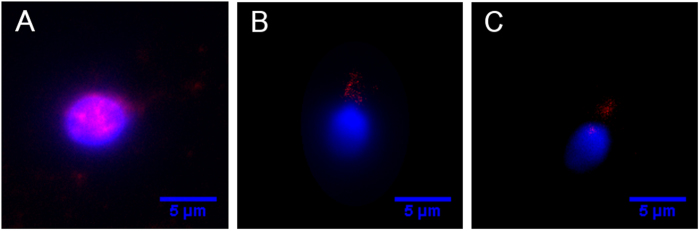
Fluorescent images of sperm stained with DAPI DNA dye and anti-γH2AX antibody. Human sperm were treated with anti-γH2AX antibody to detect double-strand breaks in DNA. Blue represents DAPI staining, indicating nuclear DNA in the head of the sperm, and red represents γH2AX staining. (**A**) Positive control. Sperm were treated with 1 mM H_2_O_2_ for 2 hours. The red areas overlaying the blue indicate the presence of double-stranded DNA breaks in the nucleus. (**B**) Experimental group. Sperm were exposed to 633 nm light for 30 minutes. There were only a few γH2AX foci seen in the sperm nuclei. (**C**) Negative control. There were little or no γH2AX foci seen in the sperm nuclei.

**Figure 4:**
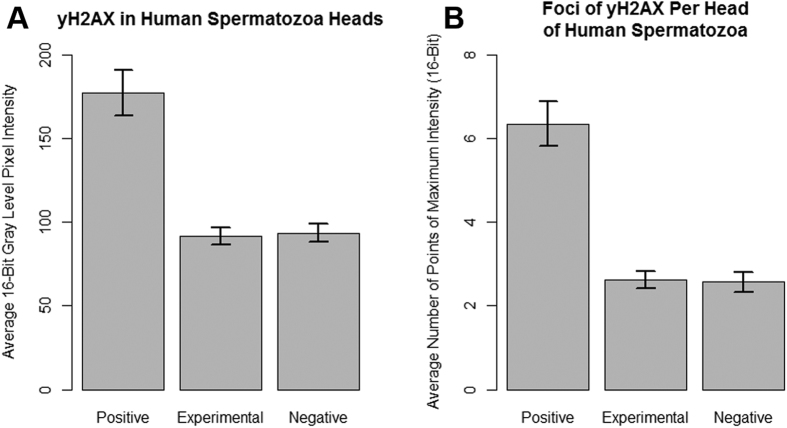
Quantification of double-strand DNA breaks in sperm using γH2AX. The mean pixel intensities and number of foci were determined using ImageJ software. Error bars indicate standard deviation. Data is representative of n = 82, n = 127, and n = 88 sperm heads for the positive, experimental, and negative groups, respectively. (**A**) A Kruskal-Wallis test determined that there was no statistical significance between the mean pixel intensities of the experimental and negative control groups (p = 0.6605). The mean pixel intensity of the positive control group was determined to be significantly higher than that of the experimental and negative control groups (p = 2.144 × 10^−10^). The mean pixel intensities for the positive, experimental, and negative groups were 177.4, 93.5, and 91.9, respectively. (**B**) A Kruskal-Wallis test indicated that the positive control had much larger numbers of γH2AX foci than the other two groups (p = 1.474 × 10^−10^). The numbers of γH2AX foci in the heads of sperm in the experimental group and negative control group were compared using a Kruskal-Wallis test (p = 0.723). The average number of γH2AX foci in the positive, experimental, and negative groups were 6.4, 2.6 and 2.6, respectively.

Fluorescent images show localization of γH2AX in each of the test groups (Fig. 3). The negative control and laser-irradiated sample did not have a statistically significant difference (p > 0.05) in the amount of γH2AX foci as determined by a Kruskal-Wallis test (Fig. 4B). The positive control had statistically significant (p < 0.01) increases of γH2AX in human sperm heads compared to the irradiated sample and the negative control as determined by a Kruskal-Wallis test (Fig. 4A).

**Figure 5:**
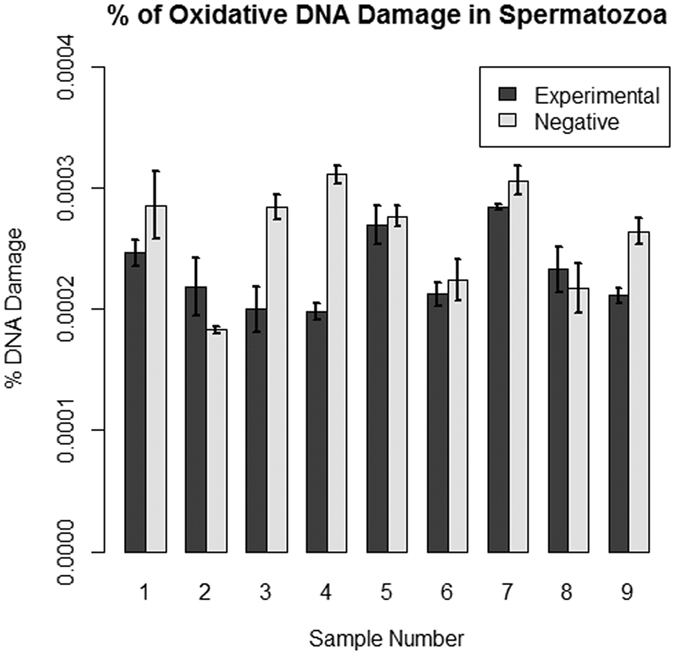
Quantification of oxidative DNA damage using 8-oxo-G ELISA. The concentrations of oxidatively damaged DNA were determined using a quantitative ELISA. Error bars represent the standard error. A Welch’s t-test determined the differences in these data were not statistically significant (p > 0.05).

A quantitative 8-OHdG ELISA was used to test for oxidative DNA damage. 9 independent sperm samples were tested for the presence of oxidative DNA damage at a concentration of 15 ng/μL. The percentages of DNA damage relative to the total DNA concentration are given in Fig. 5. The difference in calculated concentrations of damaged DNA between the two test groups in each sample was not significantly significant. Although oxidative damage was present in the samples, it makes up a small amount of the total DNA assayed. The percentage of oxidative DNA damage in both samples was about 0.0003%.

## Discussion

This study indicates that 633 nm laser irradiation of sperm at a power density of 5.66 mW/cm^2^ increases sperm swimming speed. This data supports the proposed chemical mechanism of intracellular photonic absorption, indicating that the photonic energy in red light is absorbed by cytochrome-c oxidase (Cco), increasing ATP production, and thus increasing sperm motility. In previous studies, the absorption spectra obtained for Cco in different oxidation states were recorded and found to be very similar to the action spectra for biological responses to light^16^. Therefore it was proposed that Cco is the primary photo-acceptor for the red-NIR range in mammalian cells^16^,^17^. Photonic absorption in mitochondrial Cco boosts ATP production and energy supply and may increase mitochondrial Ca^2+^ uptake. In sperm, it is believed that this results in increased motility and increased fertilization potential^18^. However, our study did not account for the possibility of increased motility due to local heating. The sperm and surrounding media may have been heated by exposure to laser light. Assuming the absorption coefficient of water at 633 nm is 0.00291 cm^−1^ the temperature rise due to laser illumination should be less than 2 × 10^−4^ degrees, which is insufficient for affecting motility.

Tracking micron-sized objects under coherent illumination is a difficult task. Due to the constantly moving fringes produced over the entirety of the image, algorithms that rely on shape or intensity information often fail. The wavelet-based approach we have developed is a useful option especially under coherent illumination. The algorithm has some drawbacks, such as increased complexity and tendency to fail for completely dead sperm. However, for most cases it is remarkably robust and is certainly comparable to the conventional CASA systems, which are relatively costly. In addition, our algorithm allows for the tracking of sperm during laser irradiation allowing the observation of the direct effects of irradiation on swimming speed. This capability eliminates the lag period during which sperm are removed from the illumination source.

To be a viable fertility treatment, it is necessary that laser irradiation does not induce DNA damage in sperm. While past studies confirm that red light increases fertilizing potential, its effect on DNA integrity has not been fully assessed. Table 1 below indicates past studies of the effect of red light on spermatozoa quality and outlines the irradiation wavelength used, the species of the sperm analyzed, and the assay used to test sperm quality. While many studies assessed whether spermatozoa were still viable following irradiation, few looked specifically at its effect on DNA integrity.

DNA damage in spermatozoa has been associated with impaired fertilization, as well as with developmental problems and difficulties with embryonic implantation. Additional problems linked to DNA damage in sperm include miscarriage and high morbidity rates of offspring^43^. A cell’s inability to cope with ROS has been linked to both DNA damage and a reduction in sperm motility^44^. The mitochondria are largely responsible for producing ROS within sperm cells and are thus likely to be responsible for causing oxidative stress. Our study demonstrates that sperm exposure to 633 nm laser light at a power density of 31 mW/cm^2^ does not cause significant levels of double-strand DNA breaks, marked by γH2AX, nor did it induce significant levels of oxidative DNA damage, measured with 8-oxo-G. H_2_O_2_ causes the indirect formation of γH2AX, which can be detected 1–2 megabases from the break site^14^. We felt that the ability of this marker to spread would allow us to more easily detect DNA damage than the more widely used sperm chromatin structure assay (SCSA), which measures DNA fragmentation. SCSA assesses DNA integrity using heat-induced or acid-induced denaturation. Damaged DNA is more susceptible to denaturation at high temperatures and at low pH conditions than intact DNA^42^. However, the source of DNA damage cannot be identified with this method. Testing for γH2AX is a highly sensitive method for detecting DNA damage caused by the production of ROS and oxidative stress in sperm. We observed high levels of γH2AX in the sperm head when exposed to H_2_O_2_, but little or no γH2AX in the laser-irradiated or negative control groups. Some antibody binding is present in the sperm midpiece, where the mitochondria are concentrated. However, the mitochondrial DNA lack the H2AX histone. Therefore, it is likely that this is an effect of non-specific antibody binding, rather than an indication of the presence of double-strand breaks.

From the quantitative ELISA, it is evident that DNA damage was detected in the experimental and negative control groups. Similar levels were detected between the two, indicating that this is likely an artifact of centrifugation, cryopreservation, and thawing. There is some variation in the amount of damaged DNA present between samples. This is the result of variation in the quality of sperm from sample to sample (each sample was taken from a different ejaculate). In all cases, the amount of DNA damage detected was miniscule compared to the amount of DNA assayed. From this data, it is likely that laser irradiation did not result in high enough levels of ROS production to cause significant DNA damage. By immunostaining with γH2AX and assaying for 8-oxo-G, we have determined that red light irradiation does not produce significant levels of oxidative damage in nuclear sperm DNA.

In order to further determine the safety of red light irradiation as a sperm motility treatment, additional DNA damage tests must be conducted. An ROS probe can be used to detect the production of ROS in the mitochondria as a result of laser irradiation. Tracking ROS may give insight into DNA repair pathways in sperm as well as ROS-induced DNA damage. Sperm DNA fragmentation assays, such as acridine orange, should be repeated in order to compare our results with those from previous studies^2^. Additionally, the proposed methodology and DNA damage analysis should be tested on fresh samples from men with poor sperm quality.

## Conclusion

Of particular interest, and novelty of this study is the application of a novel wavelet analysis algorithm. This software, which is as accurate and less costly than CASA systems, allows for more rapid analysis of the sperm motility, and removes the passivity of the system itself, exposing the sperm to light that may impact the motility measurement.

The current study supports the notion that exposure to red light can improve sperm motility while producing little or no DNA damage to sperm cells. We note that since sperm morphology and energetics are significantly different between species, the effect of photo-stimulation could also vary significantly between species. Our results open up the possibly that red light exposure could be used to improve IVF results in humans and possibly in animals obviating the need for drug induced stimulation of sperm. The current technique could also be used in conjunction with established IVF techniques to improve treatment outcomes.

## Methods

### Sample preparation

Cryogenically frozen human sperm samples were obtained from the Infertility, Gynecology, and Obstetrics Medical Group (San Diego, CA). Samples were collected from healthy men and frozen according to a standard human freezing protocol^45^,^46^. The samples were thawed in a water bath at 37 °C and centrifuged for 10 minutes at 208 g (Beckman Microfuge 12) and the pellet was resuspended in 1 mL modified human tubal fluid (HTF) (Irvine Scientific) with 5% serum substitute supplement (SSS) (Irvine Scientific). The samples were washed once more in HTF with 5% SSS. The final concentration was 30000 sperm/mL. Samples were plated in Number 1.5 glass-bottom dishes (35 mm) (Cell E & G).

### Red light stimulation

Previous work in low-level light therapy involving a 633 nm light source (Intense 7404) observed cellular responses at a power density of 5.57 mW/cm^2 ^5. To assess the effect of red light irradiation on sperm motility, we irradiated sperm for 35 minutes from above using a monochromatic, coherent 633 nm laser (Intense 7404) at an intensity of 5.66 mW/cm^2^ coupled to a multimode fiber with beam homogenizer (Medlight FD) to ensure uniform exposure. To test the effect of red light on sperm DNA, sperm were irradiated for 30 minutes from above using a 633 nm light source at an intensity of 31 mW/cm^2^ coupled to a multimode fiber with beam homogenizer. For maximum power, the laser was held at 4.65 cm above the sample to illuminate only the dish area. This distance was determined using trigonometric calculations involving the fiber optic cable frontal light distributor’s half angle of divergence and the dish diameter.

To minimize the effects of temperature variation on sperm motility, all experiments were conducted at a temperature of 37 °C and in the laboratory atmosphere,. Any heat energy transferred from the laser source to the samples was insufficient to cause temperature increases, near or at the sample surface, as measured with an infrared thermometer (Raytek Raynger MX2). To account for stray light that might affect ATP production within mitochondria, all experiments were conducted in the absence of external lighting (sunlight, laboratory lighting, etc.).

### Wavelet-based tracking algorithm

We have developed a wavelet-based tracking algorithm to measure sperm swimming speed directly under the influence of red light. Using a simple wavelet analysis, “likely” pixels that are varying within a predefined set of frequency parameters can be identified. A stack of images can be acquired under any illumination source and input into the algorithm. The wavelet analysis sorts through a stack of images based on frequency to differentiate between sperm (high-frequency information) and background (low-frequency information). The spatially localized groups of these pixels can then be tracked using a Kalman filter. Thus, sperm are tracked by their movement characteristics rather than by their shape. Below we give a pseudocode representation of the algorithm used to accomplish this.
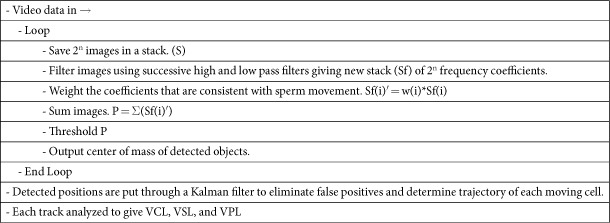


Values for average curvilinear velocity measured by our tracking program were compared to those measured by the IVOS CASA system (Hamilton Thorne) for 30 frames in a given area at a frame rate of 60 Hz. Two 6 μL aliquots were drawn from the same sample and pipetted into 20 μm 2 chamber slides (Leja). VCL measurements of sperm cells in each aliquot were taken on both systems simultaneously at random image locations. The mean VCL of all sperm cells in a given image location were averaged across all captured image locations to yield an average VCL value for that time frame (1–2 minutes). Thus, both machines output an average VCL value over the same duration of time. This process was repeated at subsequent time durations throughout the remainder of the sample’s lifetime. A total of 14 trials from 4 different specimens were conducted, with each trial representing a VCL value averaged over 1–2 minutes.

For average curvilinear velocity measurements under red light, sperm samples were plated in 35 mm dishes. VCL was measured using our wavelet algorithm. 5 trials were conducted; with each trial representing the averaged VCLs of 4 aliquots taken from a single specimen imaged 1–2 minutes apart. Samples from 2 different donors were used.

### Imaging

**Figure 6:**
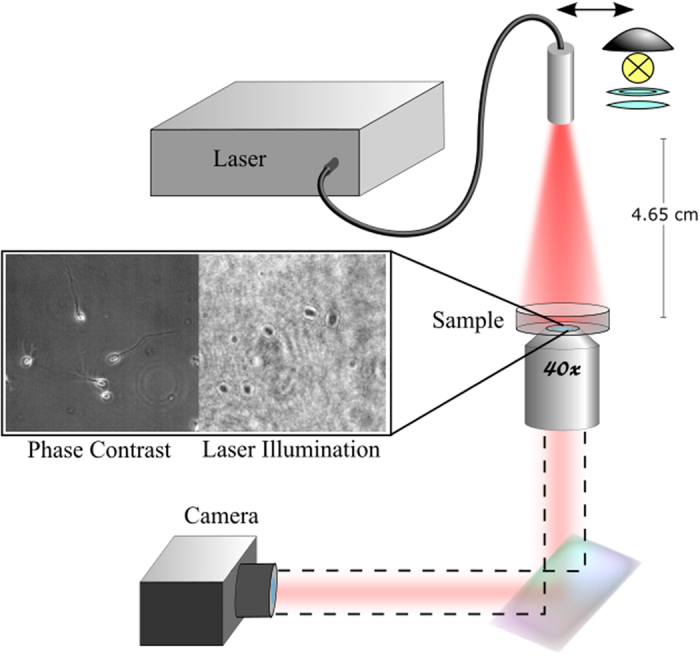
Setup of laser irradiation system. Phase contrast images were taken under the microscope’s halogen lamp. During irradiation, the sample was imaged under laser illumination (without the microscope’s halogen lamp). The light source was a fiber coupled diode laser coupled to a multimode fiber with a built-in beam homogenizer.

A Zeiss Axiovert S100 2TV microscope with a 40x, NA 1.3 phase III, oil immersion objective and Cohu 7800 CMOS camera were used for live imaging of the samples (Fig. 6). Double-stranded DNA damage was assessed via fluorescent imaging using a Zeiss microscope with a 63x, NA 1.4 phase III, oil immersion objective and a Hamamatsu ORCA-flash 4.0 CMOS camera.

### DNA damage detection

To test for DNA damage, a positive control was established by exposing live sperm to 1 mM H_2_O_2_ (Sigma) in modified HTF for 2 hours. Three 50 μL sperm samples (1 exposed to H_2_O_2_, 1 exposed to laser light, 1 untreated negative control) were plated on poly-d-lysine dishes (MatTek) and dried following exposure. The samples were then fixed with 4% paraformaldehyde in phosphate buffered saline (PBS) for 1 hour and permeabilized with 500 μL blocking buffer for 1 hour. Blocking buffer was prepared using 5% horse serum and 0.2% Triton-X in PBS. The samples were stained with 1:1000 mouse anti-γH2AX antibody (Millipore) in blocking buffer for 1 hour and incubated with 1:2000 anti-mouse secondary antibody in blocking buffer for 1 hour. The samples were stained with 1:600 DAPI (Invitrogen) in PBS for 10 minutes before imaging. The mean pixel intensity of γH2AX in the sperm head was assessed using ImageJ software (version 1.50a). γH2AX foci formation were also quantified utilizing ImageJ software.

Oxidative DNA damage was further quantified using an 8-OHdG ELISA following the manufacturer’s protocol (Cayman Chemical). Genomic DNA was extracted using a DNA isolation kit (Zymo) following the manufacturer’s protocol. This assay acts competitively; acetylcholinesterase linked to tracers are added and compete with the sample DNA to bind to the antibodies and unbound DNA is washed off. An enzymatic reaction is triggered to produce a signal from the tracer to indicate the level of bound DNA. Samples of irradiated sperm and an untreated negative control were prepared for comparison. Sperm DNA were assayed at concentrations of 1.5 × 10^7^ pg/mL in triplicate. Concentrations of damaged DNA were calculated using a standard curve. The standard curve was prepared by assaying 8 samples of known 8-oxo-G concentration (between 10.3 pg/mL and 3000 pg/mL) in duplicate.

### Statistical analysis

A post-hoc power analysis conducted in GPower 3.1.9.2 shows that a 3-group sample with n = 297 is sufficient to produce an effect size of 0.25 and α error probability of 0.05 with a power of 0.977. Statistical significance was determined using α = 0.05, where p < 0.05 indicates a less than 5% chance the test groups originated from the same population. A Shapiro-Wilk test indicated that the data used in this study were not normally distributed (p < 0.05). Thus, significance tests for non-normally distributed data were used. A Kruskal-Wallis one-way analysis of variance was used to determine whether there was a significant difference in γH2AX intensities and whether there was a significant difference in γH2AX foci formation. A Welch’s t-test was used to determine 8-oxo-G concentration between the experimental and negative control groups. Statistical analysis was done using R software (version 3.2.0).

## Additional Information

**How to cite this article:** Preece, D. *et al*. Red light improves spermatozoa motility and does not induce oxidative DNA damage. *Sci. Rep.* **7**, 46480; doi: 10.1038/srep46480 (2017).

**Publisher's note:** Springer Nature remains neutral with regard to jurisdictional claims in published maps and institutional affiliations.

## Figures and Tables

**Table 1:** Prior Work on Irradiation of Spermatozoa with Visible and IR Light.

Sperm Quality Analysis	Species Tested	Irradiation Wavelength
cell viability assay (membrane integrity)	^†^bull^19^,^20^; ^†^chicken^21^; ^†^pheasant^21^; ^†^turkey^21^; ^†^ram^22^; ^*^boar^3^; ^*^turkey^21^; ^*^rabbit^23^; ^*^bull^24^	600–700 nm
	^*^human^25^,^26^	700–1064 nm
hypo-osmotic swelling test	^†^chicken^21^; ^†^pheasant^21^; ^†^turkey^21^; ^*^human^27^; ^*^dog^28^	600–700 nm
	^*^human^29^	800–1064 nm
ROS production	^*^ram^30^; ^*^tilapia^30^; ^*^human^31^	400–800 nm
	^†^bull^20^; ^*^ram^30^; ^*^tilapia^30^	600–700 nm
sperm chromatin structure assay	^*^human^2^	905 nm
aniline blue staining	^*^human^27^	636.6 nm
sperm chromatin dispersion	^*^human^29^	830 nm
none	^*^buffalo^32^	532 nm
	^†^bull^18^,^33^; ^†^human^34^; ^*^human^34^,^35^,^36^; ^*^mouse^4^,^37^; ^*^dog^38^	600–700 nm
	^†^bull^33^,^37^; ^*^human^35^,^39^	700–1064 nm

Sperm quality analysis tests listed above reflect tests of physical defects in sperm DNA and plasma membranes. Cell viability assays and hypo-osmotic swelling tests assess membrane integrity. Aniline blue staining assesses sperm chromatin condensation through the detection of lysine residues on sperm DNA^40^. The sperm chromatin dispersion test is used to measure DNA fragmentation based on chromatin morphology^41^. The sperm chromatin structure assay (described below) measures DNA fragmentation based on susceptibility to denaturation42. †frozen specimens; *fresh specimens.
